# Rotavirus C: prevalence in suckling piglets and development of virus-like particles to assess the influence of maternal immunity on the disease development

**DOI:** 10.1186/s13567-019-0705-4

**Published:** 2019-10-22

**Authors:** Juliet Chepngeno, Annika Diaz, Francine C. Paim, Linda J. Saif, Anastasia N. Vlasova

**Affiliations:** 1 Food Animal Health Research Program, Ohio Agricultural Research and Development Center, College of Food, Agricultural and Environmental Sciences, Department of Veterinary Preventive Medicine, The Ohio State University, Wooster, OH USA; 2 Present Address: College of Food, Agricultural and Environmental Sciences, The Ohio State University, Agricultural Administration Building, Columbus, OH 43210 USA

## Abstract

Rotavirus C (RVC) has been detected increasingly in humans and swine in different countries, including the US. It is associated with significant economic losses due to diarrheal disease in nursing piglets. In this study we aimed: (1) to determine the prevalence of RVC in healthy and diarrheic suckling piglets on US farms; and (2) to evaluate if maternal antibody (Ab) levels were associated with protection of newborn suckling piglets against RVC. There was a significantly higher prevalence (*p* = 0.0002) of litters with diarrhea born to gilts compared with those born to multiparous sows. Of 113 nursing piglet fecal samples tested, 76.1% were RVC RNA positive. Fecal RVC RNA was detected in significantly (*p* = 0.0419) higher quantities and more frequently in piglets with diarrhea compared with healthy ones (82.5 vs. 69.9%). With the exception of the historic strain Cowden (G1 genotype), field RVC strains do not replicate in cell culture, which is a major impediment for studying RVC pathogenesis and immunity. To circumvent this, we generated RVC virus-like particles (VLPs) for Cowden (G1), RV0104 (G3) and RV0143 (G6) and used them as antigens in ELISA to detect swine RVC Abs in serum and milk from the sows. Using RVC-VLP Ab ELISA we demonstrated that sows with diarrheic litters had significantly lower RVC IgA and IgG Ab titers in milk compared to those with healthy litters. Thus, our data suggest that insufficient lactogenic protection provided by gilts plays a key role in the development of and the increased prevalence of clinical RVC disease.

## Introduction

Rotavirus is one of the most important etiological agents of severe diarrhea in humans, animals and birds worldwide [1]. The name rotavirus is derived from the Latin word “Rota” (wheel) because of their wheel-like appearance under the electron microscope (EM) [2]. RVs are associated with more than 100 million infections in humans, and 2 million hospitalizations and 210 000 deaths annually in children < 5 years old [3]. In animals, RV infections result in slow growth, lethargy and mortality, causing significant losses to farmers [4, 5].

RVs are triple-layered, icosahedral, non-enveloped viruses that belong to the family *Reoviridae.* Their genome consists of 11 segments of double-stranded RNA (dsRNA) encoding six structural proteins namely: VP1, VP2, VP3, VP4, VP6 and VP7 and six nonstructural proteins (NSP): NSP1, NSP2, NSP3, NSP4 and NSP5 or NSP6 depending on the translated open reading frame [6, 7]. Within the RV genus, ten genogroups/serogroups (A–J) have been identified to date based on the molecular and antigenic characteristics of VP6, the inner capsid protein. RVAs, RVBs and RVCs infect humans and a wide range of mammals, RVDs infect chickens and turkeys, RVEs infect pigs only, RVFs and RVGs infect chickens, and RVHs infect both humans and pigs [8, 9]. RVIs and RVJs were recently detected in dogs in Hungary and in bats in Serbia, respectively [10, 11].

Five of the ten RV genogroups have been detected in swine. These genogroups are RVA, RVB, RVC, RVE and RVH [12, 13]. Porcine RVC is common in contaminated environments and normally is spread by acutely or subclinically infected animals shedding the virus via feces. RVC replicates in mature enterocytes near the tips of the villi, causing diarrhea [14]. Previously known as pararotavirus, porcine RVC was first detected in a 27-day-old piglet with diarrhea from a herd in Ohio in 1980 [12]. RVC was initially thought to cause only sporadic diarrhea outbreaks in swine, but recent studies have shown that its prevalence is much higher than previously estimated, especially among nursing, 1- to 10-day-old piglets, causing significant economic loses to farmers and the pork industry [15, 16]. Currently, RVC is a major cause of gastroenteritis in neonatal (< 1 week of age) piglets. Additionally, recent studies utilizing molecular diagnostic techniques revealed an increase in the prevalence of porcine RVC in pigs of different ages, with or without diarrhea in the US, Canada, Brazil and Europe [15–20].

Similar to RVAs, a complete genome classification system based on nucleotide sequences was established for RVCs [21]. The introduced system allocates a specific genotype to each of the 11 RV genome segments according to established 85% nucleotide cut-off where VP7-VP4-VP6-VP1-VP2-VP3-NSP1-NSP2-NSP3-NSP4-NSP5/6 genes of RV strains are classified as Gx-Px-Ix-Rx-Cx-Mx-Ax-Nx-Tx-Ex-Hx where x is an integer starting from 1 onward.

Piglets are born immunocompetent and their mucosal immune system is equipped with innate immune defense mechanisms, which provide the first line of defense against pathogens [22]. However, they are agammaglobulinemic at birth and their adaptive immune system is immature. Thus, they cannot rapidly mount protective immune responses against infections including RV [23, 24] and rely on protection by colostrum and milk-derived Abs during early life [25]. Immunoglobulin G (IgG) is the most prevalent isotype in colostrum and protects against systemic infections, while secretory IgA (sIgA) is prevalent in milk and is associated with the local (mucosal) defense system [26–28]. Isotype switching from IgM to IgA occurs mainly in the germinal centers in the gut-associated-lymphoid tissue (GALT). The transfer of IgA plasmablasts from the gut to the mammary gland and the eventual release of sIgA into milk in swine is documented but not well understood [29–31]. Although some knowledge of lactogenic immunity against RVA and enteric coronaviruses was generated using porcine and rodent animal models [32–37], lactogenic protection of piglets against RVC has not been evaluated. In contrast to RVA, RVC pathogenesis is poorly understood and the role of maternal passive immunity in alleviating the clinical disease not been studied. Intriguingly, although RVA diarrhea is rare in suckling piglets and becomes more prevalent after weaning, suckling status does not seem to mitigate RVC diarrhea in piglets. However, whether it is associated with insufficient levels of maternal RVC Abs or with low minimum RVC infectious dose (compared with other swine enteric viruses) is not known [15, 18–20, 38, 39].

In this study, we evaluated RVC prevalence and/or quantities in healthy and diarrheic farm piglets. Further, to understand the role of lactogenic immunity in protection against clinical RVC disease, we evaluated the association between parity, RVC maternal Ab titers and the prevalence of RVC diarrhea in piglets [40].

## Materials and methods

### Prevalence of porcine RVC

#### Sample collection and processing

Milk and blood samples were obtained randomly from 30 farm gilts/sows (*n* = 15 with diarrheic piglets and *n* = 15 with healthy piglets) (Additional file 1) from Cooper Farms (Fort Recovery, Ohio, USA). The farm is a commercial facility with multiple swine farms. Samples were collected from one farm with 5000 gilts/sows and with all-in-all-out biosecurity measures and requiring 3 days without other pig contact for admission. Diarrhea determination was as follows: Solid or pasty feces = healthy, while liquid and watery feces = diarrheic. Milk and serum were collected once from sows ranging from day 2 to day 11 after farrowing (Additional file 1). Blood was centrifuged at 2095 × *g* for 10 min to obtain serum and stored at −20 °C until use. Milk samples were filtered through a 70 μm pore filter and centrifuged at 1800 × *g* for 30 min at 4 °C to separate fat, skim milk and cell pellet portions. Fat was removed utilizing sterile plain-tipped applicators (Fisher Scientific, Hampton, NH). Skim milk was collected, centrifuged at 28 000 × *g* for 1 h at 4 °C to separate the whey that was then stored at −20 °C until tested. Rectal swabs from piglets (2–11 days, *n* = 4/L) were collected once by inserting Dacron swab 3–5 cm into the rectum and rotating it against the rectal wall several times. In total, 113 piglets’ rectal swabs, 30 sows’ milk and 30 sows’ blood samples were obtained. Annual outbreaks of viral diarrhea in suckling piglets were reported, including during the sampling period.

### RNA extraction from rectal swabs and RT-qPCR

Rectal swabs (*n* = 273) were processed by submerging the swabs into 2 mL of Minimum Essential Medium (MEM) and 1% antibiotic–antimycotic (Anti–Anti) (Life Technologies, Grand Island, NY, USA). Centrifugation was performed at 2095 × *g* for 20 min at 4 °C. Genomic RNA was extracted from rectal swab supernatants (50 µL) using the MagMAX total RNA isolation kit (Life Technologies), according to the manufacturer’s protocol. RT-qPCR was performed using One-step RT-PCR Kit (Qiagen, Germantown, MD, USA) using the primers and probe indicated (Table 1). RT-qPCR conditions were as follows: reverse transcription at 50 °C for 30 min, initial PCR activation at 95 °C for 15 min, 40 amplification cycles with denaturation at 94 °C for 1 min, annealing at 55 °C for 1 min, extension at 72 °C for 1 min and final extension at 72 °C for 10 min. RVC RNA levels in fecal samples were converted to log10 genomic equivalence (GE) using a standard curve developed using RVC VP6 gene amplicon target and RVC diagnostic primers (Table 1). RNA extraction from a validated RVC-positive sample was used as a positive control, while RNA-free water was used as a negative control.

**Table 1:** **Diagnostic primers for porcine RVC, RVA and RVB and cloning primers for porcine RVC full length structural genes, VP7, VP6, VP4 and VP2**

Target or function	Sequence	Region (nt)	Reference/source
Detection			
RVA	Forward: 5′-ACCATCTACACATGACCCTC-3′ Reverse: 5′-GGTCACATAACGCCCC-3′	963–982	[62]
RVB	Forward: 5′-GGTTTAAATAGCCCAACCGACGC-3′ Reverse: 5′GTRTTYAAATTSGTRTTTGGCGCT-3′	1–94	[19]
RVC	Forward: 5′-ATGTAGCATGATTCACGAATGGG-3′ Reverse: 5′-ACATTTCATCCTCCTGGGGAT-3′ Probe: 5′-VIC-GCG TAG GGG CAA ATG CGC ATG A-TAMRA-3′		[19]
RVC amplification			
VP7	G1 and G3 forward: 5′-ATG GTT TGT ACR ACA TTG TRC 3′ G1 and G3 reverse: 5′-GCC ACA TGA TCT TGT TTA CGC GTA T-3′ G6 forward: 5′-ATG GTT TGT ACA ACT TTG TAC AC AGT TT-3′ G6 reverse: 5′-GCC ACA TGA TCT TGT TTA CGC GTA TC-3′	1–1054	This study
VP6	G1 and G3 G6 forward: 5′-ATG GAT GTA CTT TTT TCY ATY GCG-3′ G1, G3 and G6 reverse: 5′-TTC GCC CTT AGC CAC ATA GTT CAC ATT TC-3′	1–1294	This study
VP4	G1, G3 and G6 forward: 5′-GGA TCA ATG GCG TCC TC-3′ G1, G3 and G6 reverse: 5′-AGC CAC ACA ATC AGT CGA TCT CCT CAC-3′	1–2232	This study
VP2	G1 and G3 forward: 5′-ATG ATA AGC AGG AAY AGA C-3′ G1 and G3 reverse: 5′-CAG AAT TTG AGG TCR TCA CAA GAT-3′ G6 forward: 5-ATG ATA AGC AGG AAT AGA-3′ G6 reverse: 5′-GAA TTT GAG GTC ATC ACA AGA TG-3′	1–2737	This study

RNA isolated as above was also tested for porcine RVA and porcine RVB using RT-PCR and specific primers as described by Amimo et al. [15]. Porcine epidemic diarrhea virus (PEDV) and porcine deltacoronaviruses were analyzed using conventional PCR as described in Jung et al. [41], Vlasova et al. [42] and Hu et al. [43].

### Development of RVC VLP based antibody ELISA

#### RNA extraction and amplification of porcine RVC structural genes by conventional RT-PCR

Intestinal contents of gnotobiotic (Gn) piglets inoculated with porcine RVC RV0104 G3 and RV0143 G6 genotypes [15] were diluted (1:10) in MEM media containing 1% antibiotic–antimycotic 100 Mm and centrifuged at 2095 × *g*, 4 °C for 20 min. Genomic RNA was extracted from Cowden G1 cell culture, RV0104 G3 and RV0143 G6 supernatants (50 µL) using the MagMAX total RNA isolation kit (Life Technologies) according to manufacturer’s protocol. The RNA obtained was converted into cDNA using SuperScript™ III Reverse Transcriptase and random hexamers as primers (Thermofisher Scientific, Waltham, MA, USA). cDNA synthesis was performed in the following condition: 50 °C for 50 min, 85 °C for 5 min. RNase H was added and incubated at 37 °C for 20 min to remove RNA. To amplify RVC structural genes, Takara PrimeSTAR GXL Polymerase kit (TakaraBio, Mountain View, CA, USA) and primers (Table 1) were used to amplify RVC structural genes. The reagents used were as follows; 5 μL of 5× PrimeSTAR GXL Buffer, 2 μL 1× dNTP Mixture (2.5 mM each), 2 μL of reverse and forward primers (Integrated DNA Technologies, Inc. Skokie, Illinois, USA), 1 μL of RNAsin (Promega, Madison, WI, USA), 11 μL of RNAse free H_2_O and 2 μL of cDNA. Thermocycler conditions were set as follows; 40 cycles of 98 °C for 10 s, 55 °C or 60 °C (if primer annealing temperature is below 55 °C, it was set at 55 °C and if primer annealing temperature was above 55 °C, the temperature was set at 60 °C) for 1 min, and 68 °C for 1 min per kb extension time. Results were analyzed using 1% agarose gel.

#### Sequencing

Amplified structural genes (VP7, VP4, VP2 and VP6) were purified by QIAGEN Gel Extraction Kit (Qiagen, Hilden, Germany) as described by the manufacturer. Expected sizes of DNA fragments were excised from the 1% agarose gel and dissolved using buffer QG (3× of gel weight) at 50 °C. Once the gel dissolved, 1 gel volume of isopropanol was added and the solution was transferred into the spin column. Columns were centrifuged for 1 min at 10 000 × *g*, flow through discarded, 500 µL of buffer QG was added, centrifuged again at 10 000 × *g* at 1 min and flow through was discarded. 750 µL of buffer PE was added to columns, centrifuged at 10 000 × *g* for 1 min and flow through was discarded. The spin column was transferred to a clean 1.5 mL micro centrifuge tube and the DNA bound to the column was eluted using 50 µL of elution buffer by centrifugation at 10 000 × *g* for 1 min. PCR products along with corresponding forward and reverse primers (Table 1) were submitted to the molecular and cellular imaging center (MCIC), OARDC, Wooster, Ohio for sanger sequencing. The sequences obtained were analyzed using BLAST software from PubMed [44]. After amplification of VP4 structural genes from RVC VP7 genotypes Cowden G1, RV0104 G3 and RV0143 G6, the amplicons were sequenced, and the sequences were aligned and analyzed using MEGA 7.0 software. Phylogenetic analysis was performed using MEGA 7.0 software and Bootstrap method (1000).

#### Cloning

Amplification primers (Table 1) were designed with the additional sequence (CACC) on the 5′ end to enable directional cloning into pENTR/D/TOPO (Life Technologies). Amplification products of the structural genes (1 μL) were combined with One Shot^®^ Chemically Competent *E. coli* (Life Technologies) and heat shocked for 30 s at 42 °C without shaking. Super optimal broth medium (250 μL) (Life Technologies) was added and incubated in a 37 °C water bath for 1 h with shaking. The bacterial culture was plated on a pre-warmed 40 ng/mL kanamycin selective plate and incubated overnight at 37 °C. Colonies were selected and grown in Luria–Bertani (LB) media with 40 ng/mL kanamycin and incubated overnight at 37 °C with shaking. Purification of plasmids was performed using PureLink™ 96 HQ Plasmid DNA Purification Kit (Life Technologies) following the manufactures’ instructions.

#### Generation of recombinant baculovirus DNA

To transfer genes of interest from pENTR/D/TOPO^®^ gateway entry vector, BaculoDirectTM C-Term linear DNA (Life Technologies) and recombinant pENTR/D/TOPO^®^ gateway plasmids were recombined according to the manufacturer’s instructions. The transfer of the genes was confirmed using RT-PCR with Polyhedrin forward primer (5′-AAATGATAACCATCTCGC-3′) and V5 Reverse Primer (5′-ACCGAGGAGAGGGTTAGGGAT-3′). RT-PCR products were screened using 1% agarose gel.

#### *Spodoptera frugiperda* 9 (Sf9) cells growth and maintenance

Sf9 cells were obtained from Invitrogen and maintained in Gibco^®^ Sf-900™ II serum free media (Life Technologies, supplemented with 10% heat-inactivated fetal bovine serum (Atlanta Biologicals, Flowery Branch, GA, USA) and 1% Penicillin Streptomycin mixtures contain 5000 units of penicillin (base) and 5000 µg of streptomycin (base)/mL utilizing penicillin G (sodium salt) and streptomycin sulfate in 0.85% saline (Life Technologies). The cells were grown in spinner flasks in a 27 °C incubator and cell concentrations were maintained between 0.7 × 10^6^ cells/mL and 2 × 10^6^ cells/mL.

#### Transfection of insect cells and expression of recombinant protein

Sf9 cells were seeded into a 6-well tissue culture plates at 8 × 10^5^ cells/well with 2 mL of non-supplemented Grace’s insect Medium (Life Technologies) without antibiotics and serum and then incubated for 30 min at 27 °C to allow the cells to fully attach to the bottom of the plate. Recombinant baculovirus DNAs containing the RVC genes were transfected into Sf9 cells using Cellfection^®^ II Reagent (Life Technologies). The cells were incubated at 27 °C for ~72 h until they exhibited cytopathic effects (CPE). Then, the supernatant medium was collected from each well and centrifuged to remove cells and large debris and designated as the P1 viral stock.

To express recombinant protein, Sf9 cells were seeded in 6-well tissue culture plates at 2 × 10^5^ cells/well with 2 mL of Gibco complete growth medium (Life Technologies) and incubated at 27 °C for 30 min to allow the cells to fully attach to the bottom of the plate. P1 stock (20 µL) was used to infect Sf9 cells to generate a higher protein expression and designated as P2 stock that was used to generate the P3 stock following manufacturer’s protocol (Life Technologies). Cell lysates were monitored for RVC protein expression by Western blot. Cowden G1 hyperimmune serum obtained from a Gn pig was used as primary Ab and anti-swine IgG HRP conjugate was used as secondary Ab (Bio-Rad, Berkeley, CA, USA).

#### VLP expression, purification and visualization

Six combinations of RVC core and outer capsid proteins were produced (Table 2). The combinations of core and outer capsids of P3 viral stock were used to co-infect Sf9 cells at MOI 5 and incubated at 27 °C. Infected Sf9 cells were harvested at day 7 by centrifuging at 4 °C, 2095 × *g* for 20 min to remove cell debris. Supernatants were further semi-purified using ultracentrifuge at 106 979 × *g* through 35% (w/v) sucrose cushion for 2 h. The pellets were recovered and resuspended in cold TNC buffer (10 mM Tris–HCl [pH 7.4], 140 mM NaCl, 10 mM CaCl_2_) and stored at 4 °C. For VLP visualization by electron microscopy (EM), 1.3 µg of semi-purified RVC VLPS was added to 11 mL of deionized filtered H_2_O and ultracentrifuged at 209 678 × *g* for 30 min. The recovered pellet was resuspended in 20 µL of deionized H_2_O. 10 µL of VLPs was added to carbon coated grid for 2 min and excess removed using filter paper. 10 µL of 2% Uranyl acetate was added to the grid for 1 min in dark. The grid was left to dry for 30 min and observed under electron microscope Hitachi H-7500. EM image of the semi-purified RVC VLP was taken under magnification 50 K using Hibachi H7500 EM.

**Table 2:** **Expressed and assembled porcine RVC VLPs**

Number	2/4/6/7 RVC structural genes combinations to form VLP
1	VP4 G3, VP7 G3, VP6 G6, VP2 G6
2	VP4 G6, VP7 G6, VP6 G6, VP2 G6
3	VP4 G1, VP7 G1, VP6 G1, VP2 G1
4	VP4 G3, VP7 G3, VP6 G1, VP2 G1 (VP6 G3, VP2 G3)
5	VP4 G1, VP7 G1, VP6 G6, VP2 G6
6	VP4 G6, VP7 G6, VP6 G1, VPG G1
7	Cocktail mixture of VLPs # 1–6

PCR was used to determine the presence of RVC VP7, VP4, VP6 and VP2 in recombinant baculovirus DNA Combinations of different genotypes (G1, G3 and G6) (1–6) shown in the table were used to co-infect Sf9 cells. After harvest and semi-purification, combinations 1–6 were used to make the cocktail of RVC VLPs #7.

### Development of RVC virus-like-particle antigen ELISA to quantitate RVC antibodies

Immune serum was obtained from RVC Cowden G1, RV0104 G3 or RV0143 G6 inoculated Gn piglets. Cowden G1 serum was collected 21 days post-inoculation. RV0104 G3 and RV0143 G6 sera were collected 10 days post-inoculation. VLP combinations (100 ng) diluted in ELISA coating buffer (0.05 M carbonate–bicarbonate, pH 9.6) as described in Table 2 were added to immunoassay plates (Life Technologies) and incubated overnight at 4 °C. Plates were washed 3× using washing buffer (50 mM Tris, 0.14 M NaCl, 0.05% Tween 20, pH 8.0). Serum (Cowden G1, RV0104 G3 and RV0143 G6) was diluted with PBS (1:100, 1:400, 1:1600 and 1:6400) and added (100 µL) to the duplicate wells of washed immunoassay plates. The plates were incubated at 37 °C for 1 h and washed 3× using wash buffer. Anti-swine IgG conjugated to horseradish peroxidase (1:250 in PBS, 100 µL) (Bio-Rad, California, USA) was added and incubated at 37 °C for 1 h, then plates were washed 3× with washing buffer and 3,3′,5,5′-tetramethylbenzidine substrate (Life Technologies) (100 µL) was added. The reaction was stopped with 1 M hydrochloric acid after 15 min. The plates were read using an ELISA microplate reader (Molecular Devices SPECTRAMAX 340 PC 384, Sunnyvale, California, USA) at 450 nm. The ELISA Ab titer was expressed as the reciprocal of the highest dilution that had a corrected *A*450 value (sample absorbance in the RVC positive control serum samples (serum collected from Gn piglets infected with RVC) wells minus sample absorbance in RVC negative serum (serum collected from Gn piglets with no virus) greater than the cut-off mean corrected *A*450 value of negative controls plus 3 standard deviations).

### Screening serum and milk of sows/gilts for RVC IgA and IgG antibodies using the VLP ELISA and genotype specific VLP ELISA

VLP antigens (100 ng of cocktail mixture or 100 ng of Cowden G1 VLP #3, RV0104 G3 VLP #4 or RV0143 G6 VLP #2 for genotype specific assay as indicated in Table 2) diluted in ELISA coating buffer (0.05 M Carbonate-Bicarbonate, pH 9.6) were used to coat immunoassay plates and incubated overnight at 4 °C. Plates were washed 3× using washing buffer (50 mM Tris, 0.14 M NaCl, 0.05% Tween 20, pH 8.0). Gilt/sow serum and milk were serially diluted fourfold in PBS (1:25, 1:100, 1:400 and 1:1600 for cocktail mix and 1:400, 1:1600, 1:6400 and 1:25 600 for genotype specific) and added (100 µL) to the duplicate wells of washed immunoassay plates. The plates were incubated at 37 °C for 1 h and washed 3× using wash buffer. Anti-swine IgG or IgA conjugated to horseradish peroxidase (1:250 in PBS, 100 µL) were added and incubated at 37 °C for 1 h then washed 3× with washing buffer. 3,3′,5,5′-tetramethylbenzidine substrate (100 µL) (Life Technologies) was added and reaction was stopped with 1 m hydrochloric acid (100 µL) after 15 min. The plates were read using an ELISA microplate reader (Molecular Devices SPECTRAMAX 340 PC 384, Sunnyvale, California) at 450 nm. The ELISA Ab titer was expressed as the reciprocal of the highest dilution that had a corrected *A*450 value (sample absorbance in the serum/milk samples wells minus sample absorbance in RVC negative serum (serum collected from Gn piglets with no virus) greater than the cut-off mean corrected *A*450 value of negative controls plus 3 standard deviations).

### Statistical analysis

Spearman’s rank correlation analysis was conducted to evaluate the relationship between the parity numbers and diarrhea prevalence in their piglets. The Chi-square test was used to evaluate the prevalence of RVC shedding in diarrheic and healthy piglets. Statistical analysis of virus shedding prevalence and titers and IgA/IgG Ab titers in gilt/sow milk and serum samples were done using t-test. Statistical significance was assessed at *p* ≤ 0.05 for all comparisons. All statistical analyses were performed using GraphPad Prism 7.0c (GraphPad Software Inc. CA, USA).

## Results

### Parity number was significantly negatively correlated with prevalence of diarrheic litters

The Spearman rank correlation analysis demonstrated that there was a significant negative correlation between the parity (Sr = −0.6219, *p* = 0.0002) and the diarrhea prevalence in piglets, with a majority of diarrheic litters (73%) born to gilts (Additional file 1, Figure 1A).

**Figure 1:**
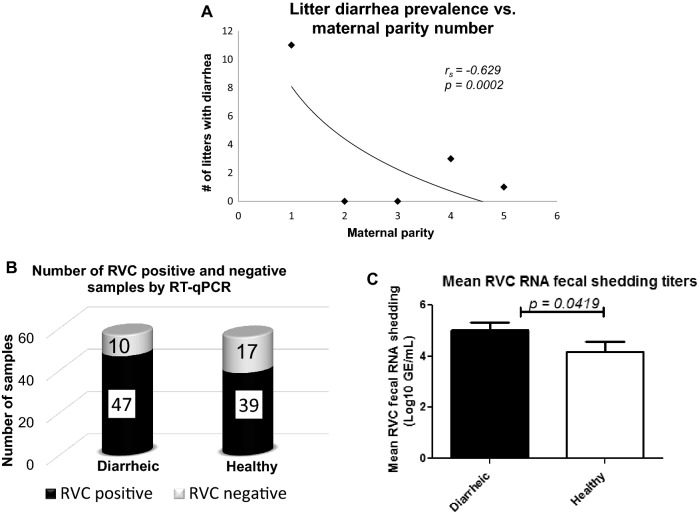
**Correlation of litters with diarrhea and maternal parity, prevalence of porcine RVC in diarrheic and healthy piglets, and piglets mean fecal RVC shedding titers. A** Rectal samples were collected from diarrheic or healthy litters (*n* = 4). The number of litters with diarrhea was plotted against maternal parity and correlation determined using the spearman rank test (r_s_ = −0.629). Gilts (1^st^ parity) were significantly (*p* = 0.0002) associated with having diarrheic piglets. **B** RT-qPCR was used to test rectal swab samples obtained from diarrheic and healthy piglets for RVC RNA. **C** CT values from RT-PCR were converted to log10 GE/mL using a standard curve. Diarrheic piglets had significantly higher (*p* = 0.0419) RVC RNA fecal titers when compared with healthy piglets.

### Prevalence of porcine group C rotavirus in suckling piglets

Of 113 farm piglets’ fecal samples tested from healthy and diarrheic piglets, 76.1% were RVC RNA positive (Figure 1B). Further, RVC RNA was detected with higher frequency (not significant, *p* = 0.11) among the samples from diarrheic (82.5%) compared with healthy piglets (69.9%) (Figure 1B). However, RVC RNA shedding titers were significantly higher in diarrheic vs. healthy piglets (*p* = 0.0419) (Figure 1C). No PEDV, PDCoV or RVB RNA was present in these samples, while RVA were present in 6.2% of these samples.

### VLP expression and purification

**Figure 2:**
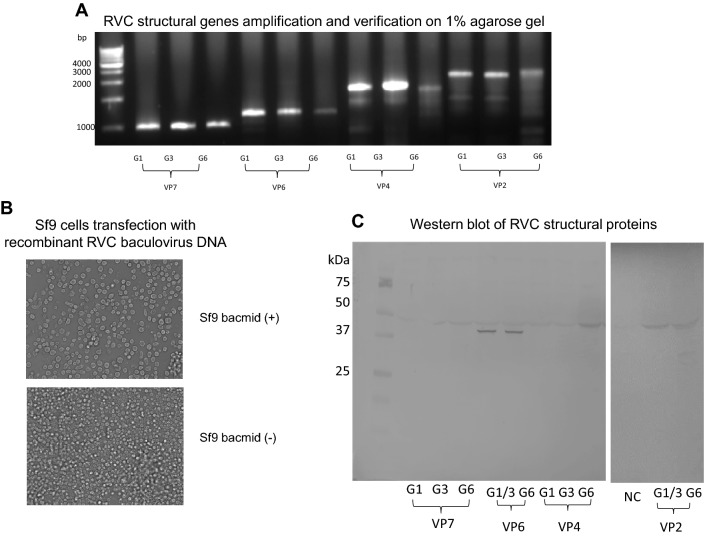
**Porcine RVC structural protein expression. A** Amplification of porcine RVC G1, G3 and G6 structural genes; VP7, VP6, VP4 and VP2 using specific primers. Amplicons were analyzed using 1% agarose gel. Expected sizes were 1054 bp for VP7, 1293 bp for VP6, 2432 bp for VP4 and 2724 bp for VP2 structural genes. **B** Sf9 cells were transfected with RVC recombinant baculovirus DNA containing individual porcine RVC structural genes (VP2, VP4, VP6, and VP7) from three strains G1, G3 and G6 at day 6 showing CPE. **C** Western blot of VP7, VP6, VP4 and VP2 protein expression using hyper immune antiserum for G1 as the primary antibody and anti-swine IgG HRP conjugated secondary antibody. The blot was developed using 3,3′,5,5′-tetramethylbenzidine (TMB) substrate.

VP2, VP4, VP6 and VP7 were amplified by conventional RT-PCR using the primer sets shown in Table 1. The sizes of the amplicons were 2724 bp, 2232 bp, 1293 bp and 1054 bp size fragments respectively, as identified on 1% agarose gel electrophoresis (Figure 2A).

Phylogenetic analysis of VP4 gene sequences of Cowden G1, RV014 G3 and RV0143 G6 strains revealed three different p-types of P1, P18 and P5 respectively (data not shown). Based on these results, we decided that VP4 genes originated from all three strains have to be used to generate VLPs.

The RVC recombinant Baculovirus DNAs were screened by RT-PCR using the primer set polyhedrin forward primer, V5 Reverse primer and with amplicons of 2967 bp, 2472 bp, 1533 bp and 1294 bp for VP2, VP4, VP6 and VP7 respectively (data not shown). The positive recombinant bacmid DNAs were transfected into Sf9 cells and cytopathic effects (CPE) were observed (Figure 2B). Expression of RVC structural proteins in RVC recombinant baculovirus stock was analyzed by Western blot. VP6 of Cowden G1/RV0104 G3 and RV0143 G6 were clearly visualized at ~ 48 kDa using hyperimmune antiserum to RVC Cowden G1 strain (Figure 2C).

**Figure 3:**
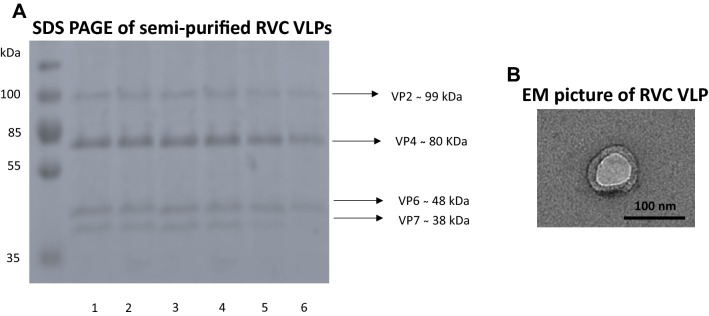
**Virus-like particles generation. A** Sf9 cells were co-infected with six different genotype (G1, G3 and G6) combinations recombinant baculovirus expressing RVC core and outer capsid protein genes shown in Table 2. At day 7 the VLPs were harvested and semi-purified through sucrose were gradient. Analysis of semi-purified VLPs was done using a 12% precast protein gel. Lanes were loaded as follows: (1) VP7 G3, VP4 G3, VP6 G6, VP2 G6. (2) VP7 G6, VP4 G6, VP6 G6, VP2 G6. (3) VP7 G1, VP4 G1, VP6 G1, VP2 G1. (4) VP7 G3, VP4 G3, VP6 G1, VP2 G1. (5) VP7 G1, VP4 G1, VP6 G6, VP2 G6 and (6) VP7 G6, VP4 G6, VP6 G1, VP2 G1. **B** EM image of porcine RVC VLP G6 genotype produced by infecting Sf 9 cells with recombinant baculoviruses containing ORFs coding for porcine RVC VP2, VP4, VP6 and VP7. Scale bar 100 nm.

Sf9 cells co-infected with different combinations of RVC recombinant baculoviruses shown in Table 2 were harvested and purified. SDS PAGE of each semi-purified VLP combination showed structural proteins VP2, VP4, VP6 and VP7 at ~99 kDa, ~80 kDa, ~48 kDa and ~38 kDa respectively (Figure 3A). EM has confirmed the presence of fully assembled triple-shelled RVC VLPs of G6 genotype (Figure 3B).

### Antigenicity of expressed RVC VLPs in ELISA to detect RVC antibodies

**Figure 4:**
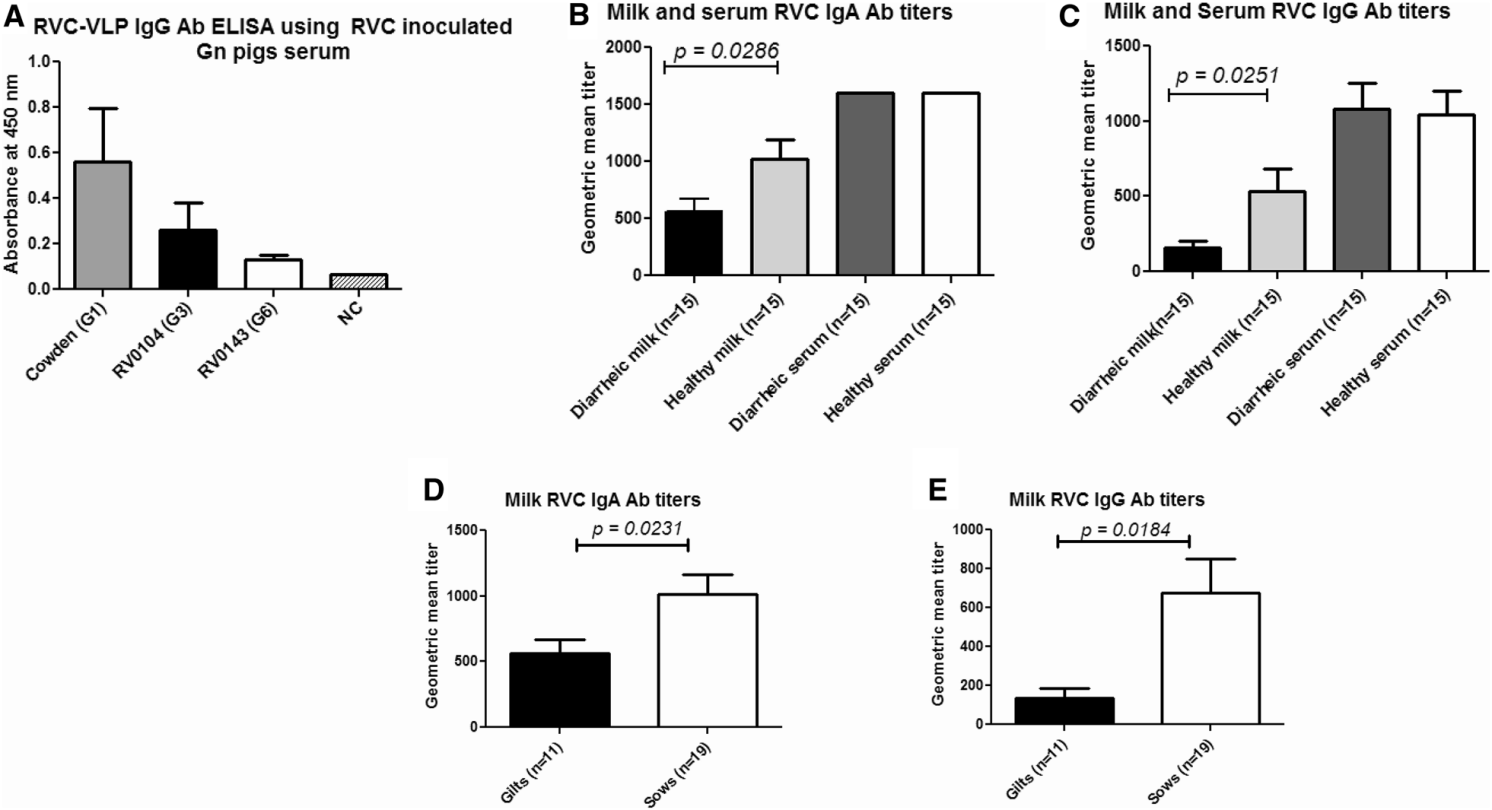
**Analysis of antigenicity of purified VLPs using RVC immune Gn piglet sera Ab ELISA and antibody titers in gilts/sows milk and serum samples. A** Antigenicity of purified RVC VLPs was tested using serum from Gn piglets inoculated with G1, G3 or G6 porcine RVC strains in a VLP-based indirect Ab ELISA. Cowden serum was collected at PID 21 while G3 and G6 serum were collected at PID 10. Negative control serum was collected prior to virus inoculation. **B**, **C** Cocktail mixture of VLPs (Table 2) was used as antigens in an RVC VLP-based indirect Ab ELISA. Milk and serum from sows/gilts with diarrheic or healthy piglets (fourfold serial dilutions 1:25, 1:400, 1:1600 and 1:6400) were tested for RVC IgA (**B**) and IgG (**C**) Ab titers. **D** Comparative analysis of milk IgA antibody titers in all sows and gilts. **E** Comparative analysis of milk IgG antibody titers in all sows and gilts.

Individual purified VLP combinations or a mixture of all VLPs (cocktail) (Table 2) were used as antigen in an indirect ELISA. Using, convalescent (Cowden G1) and acute stage (RV0104 G3 and RV0143 G6) serum samples obtained from RVC inoculated Gn piglets, we tested the antigenicity of the obtained VLPs with the various RVC antisera. Regardless of VLP combination used as antigen, we observed higher RVC Ab absorbance in Cowden G1 immune serum collected 21 days post-inoculation followed by RV0104 G3 and RV0143 G6 immune serum collected 10 days post-inoculation using VLP cocktail (Figure 4A). However, VLP combination #7 (cocktail mixture of all VLPs) gave the highest difference in absorbance at 450 nm between the Ab positive samples and the Ab negative samples and that combination was the most sensitive for all sera from Cowden G1, RV0104 G3 and RV0143 G6 RVC genotypes.

### Multiparous sows farrowed healthy litters corresponding to higher RVC IgG and IgA Ab titers in milk

In the indirect RVC VLP-based Ab ELISA, RVC milk IgA and IgG Ab titers were significantly higher (*p* = 0.0286 and *p* = 0.0251 respectively) in gilts/sows with healthy piglets compared with gilt/sows whose piglets were diarrheic (Figures 4B and C) There were no significant differences in the titers of serum IgA and IgG Abs between the two groups (Figures 4B and C). Additionally, sows’ milk IgA and IgG Abs were significantly higher (*p* = 0.0231 and *p* = 0.0184 respectively) when compared with gilts’ milk IgA and IgG Abs (Figures 4D and E).

### Levels of antibodies against the historic RVC Cowden strain were lower in milk but higher in serum

**Figure 5:**
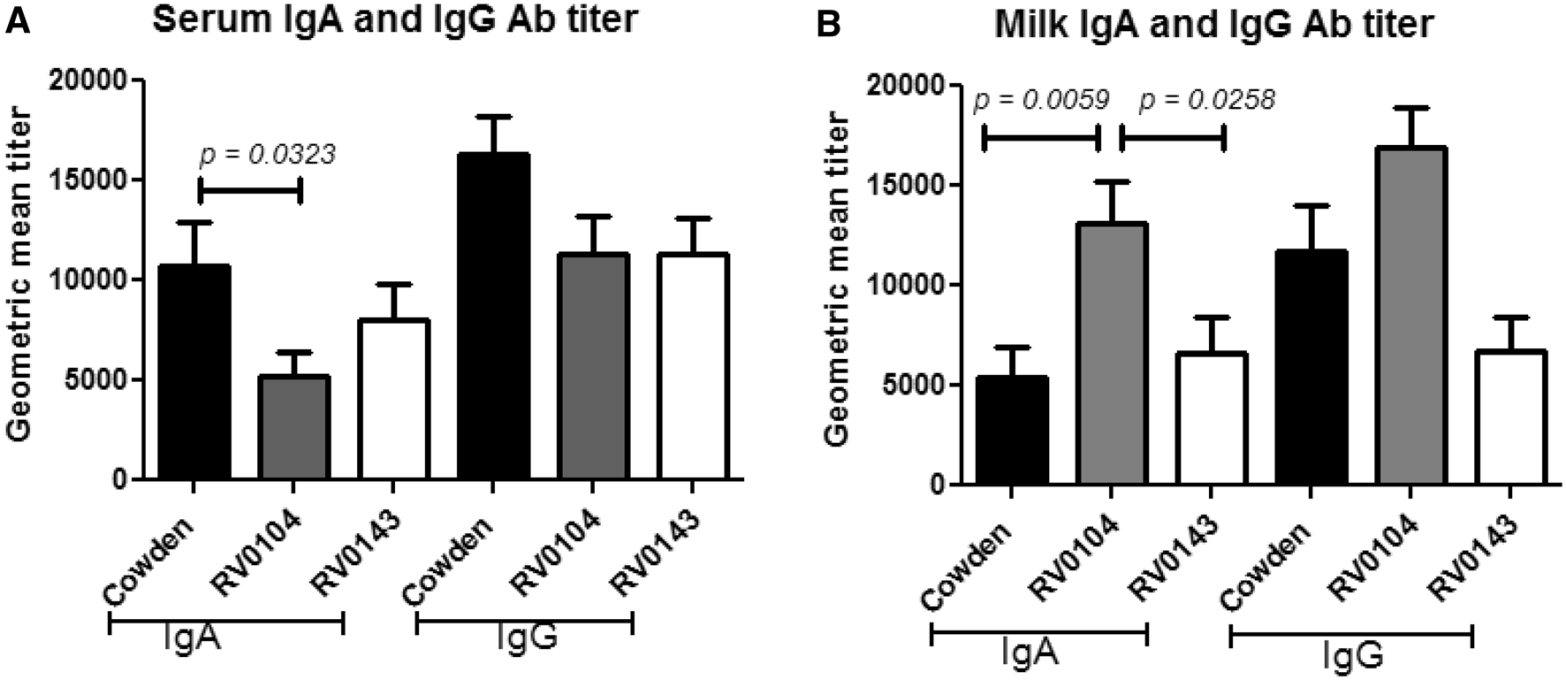
**Antibodies to the historic RVC Cowden strain are transferred less efficiently across gut-mammary axis.** Genotype specific VLPs (Cowden G1, RV0104 G3 and RV0143 G6 were used to coat immunoplates. Serum (**A**) and milk (**B**) from sows/gilts in Table 1 supplemental (fourfold serial dilutions 1:400, 1:1600, 1:6400 and 1:25 600) were tested for RVC IgA Ab titers.

Genotype specific indirect RVC VLP-based ELISA demonstrated that in serum samples Cowden-specific IgG and IgA Ab titers were higher compared with RV0143 G6 (numerically) and RV0104 G3 (significantly *p* = 0.0323) specific Ab titers. In contrast, in milk samples RV0104 G3-specific Ab titers were significantly higher compared with Cowden G1 (*p* = 0.0059) and RV0143 G6 (*p* = 0.0258) specific Ab titers (Figures 5A and B). Additionally, similar to total RVC Ab levels, there was a trend for higher genotype specific RVC Ab titers in milk and serum of sows with non-diarrheic litters (data not shown).

## Discussion

Traditionally, RVA was thought to be the major group of RVs causing diarrhea in nursing piglets. For decades, RVA epidemiology, pathogenesis and immunity were studied extensively [45–47]. However, recent studies have shown that RVC is endemic in swine worldwide and its economic importance can no longer be neglected [15, 18–20, 48, 49]. Continuous screening of RVC infections in swine is necessary to estimate RVC prevalence and to understand the protective potential of maternal RVC Abs to develop better preventive and control measures. Recent studies have shown that porcine RVC prevalence in nursing pigs is high and we hypothesized that higher prevalence would be observed in diarrheic piglets when compared with healthy piglets. Our results show that overall RVC prevalence in nursing piglets was 76.1%, in agreement with recent studies that demonstrated high RVC prevalence in nursing piglets [15, 19]. The increased RVC shedding in diarrheic piglets (82.5%) when compared with healthy piglets (69.9%) coincided with increased diarrhea prevalence suggesting its pathogenic role. Although, our results suggest that not only nursing piglets with clinical signs shed RVC RNA, but also asymptomatic nursing piglets, we observed significantly higher (*p* = 0.0419) shedding titers in fecal samples of diarrheic piglets when compared with healthy RVC positive piglets. Our finding that RVC prevalence was much higher in these samples when compared with porcine RVA and RVB is in agreement with previous data by Marthaler et al. [19] showing that porcine RVB is more often detected in older pigs whereas RVC is more frequently detected in very young piglets. Because RVA-associated diarrhea is more prevalent in older, suckling and weaned piglets, it was suggested that maternal Abs confer sufficient early protection to nursing piglets [50–53]. In contrast, RVC diarrhea prevalence was highest and increasing in nursing piglets, suggesting that RVC lactogenic protection and pathogenesis of RVC might be distinct from that of RVA and needs to be evaluated further.

To evaluate the influence of lactogenic immunity on the development of clinical RVC disease, accurate tools for serodiagnosis are required. Due to the lack of a cell culture system permissive to various RVC strains (except Cowden G1 strain) and insufficient information on pathogenesis, diversity and immunity for porcine RVC, commercial porcine RVC Ab detection ELISA assays have not been developed [54]. RVC G3 (RV0104) and G6 (RV0143) genotypes were recently identified as dominant in Ohio swine farms [15]. Additionally, Marthaler et al. [16] has recently demonstrated that G6 was the main G-genotype currently circulating in the US (70% of all RVC strains tested) G6 (70%), followed by G5 (17%), G1 (12%), and G9 (1%). In this study, we confirmed that these new circulating RVC strains (RV0104 G3 and RV0143 G6), and the historic Cowden G1 possess distinct P-types of P18, P5 and P1 respectively, suggesting that VP4 proteins from all three strains also need to be included in VLP preparations used for Ab ELISA. To our knowledge, this is the first RVC VLP-based antigen ELISA for Ab detection against the three major G-type genotype [G1 (Cowden), G3 (RV0104), G6 (RV0143)] of RVCs circulating in the US. This ELISA was further used to evaluate whether insufficient levels of maternal RVC Abs are associated with clinical disease in nursing piglets.

Because there is no transplacental transfer of immunoglobulins in swine, piglets are born agammaglobulinemic which makes availability of colostral/milk Abs critical for their survival, growth and protection against invading pathogens [24]. Because RVC is an intestinal pathogen, the presence of maternal Abs (mainly IgA) in sufficient quantities in the gut is of utmost importance. Our results show that gilts had significantly (*p* = 0.0002) higher numbers of diarrheic litters when compared with sows (multiple parity), supporting previous findings showing that piglets born to gilts’ had increased mortality rates following PEDV infection and that mortality rates declined with increased sow parity number [55]. Also, previous studies demonstrated that IgG and IgA Ab levels in serum, colostrum and milk of multiparous sows, as well as in serum of their piglets, were higher than those of first parity gilts and their piglets, respectively [56–58]. Studies of porcine RVA have shown that maternally derived Abs play a significant role in mitigating clinical disease following RV infection in neonatal swine and that the protection afforded by these antibodies is titer dependent [59]. We observed that milk IgA and IgG Ab titers were significantly higher (*p* = 0.0251 and *p* = 0.0286, respectively) in sows with healthy piglets when compared with gilt/sows with diarrheic piglets. Although some studies showed that serum IgG and IgA Abs levels were higher in sows than in gilts [56, 57], we observed no differences in RVC serum IgG and IgA Ab levels between sows with heathy piglets and sows/gilts with diarrheic piglets. Thus, as noted in studies of immunity to TGEV and PEDV [60, 61], our current results suggest that the local secretion of RVC Abs in milk, and not the levels of systemic RVC Abs contribute to piglet protection against clinical RVC disease. Lastly, we observed significantly higher IgA (*p* = 0.0231) and IgG (*p* = 0.0184) Ab titers in milk of multiparous swine when compared with gilts, suggesting that local secretion of IgA in milk or trafficking of IgA plasmablasts from the gut to the mammary glands might be influenced by the parity number [61]. Interestingly, in Cowden genotype specific ELISA, we observed higher IgA and IgG Ab titers in serum, but lower levels in milk when compared with the RV0104 G3 genotype suggesting that currently circulating RVC strains may stimulate anamnestic immune responses and gut-mammary trafficking of the infecting RVC genotype more efficiently. More studies are needed to determine the mechanisms involved in the maternal Ab transfer in milk via the gut-mammary axis.

In summary, we report that porcine RVC infections are endemic and widespread in both healthy and diarrheic neonatal piglets with higher RVC prevalence observed in diarrheic piglets. In addition, we have developed the first RVC multi-genotype VLP-based Ab ELISA for quantitation of RVC Ab. Further, we used this ELISA to measure the levels of RVC IgA and IgG Abs in sow/gilt milk and serum samples and established that lower levels of milk IgA/IgG Abs were strongly associated with the development of clinical disease and the increased RVC shedding in piglets. Finally, our results suggest that piglets born to gilts vs. multiparous sows are at higher risk of diarrheal disease induced by RVC and possibly other pathogens. Thus, our findings provide new knowledge relevant to the management of infectious diarrhea in piglets to reduce its impact on the swine industry.

## Supplementary information

**Additional file 1. Summary of farm swine herd sample collection data.**

## Data Availability

The datasets during and/or analysed during the current study available from the corresponding author on reasonable request.
