# Supplementary material for: Rotavirus C: prevalence in suckling piglets and development of virus-like particles to assess the influence of maternal immunity on the disease development
Source: Vet Res. 2019 Oct 22;50:84. doi: 10.1186/s13567-019-0705-4 (PMC6805359; doi:10.1186/s13567-019-0705-4)
Supplement: Supplementary file 1 — Additional file 1. Summary of farm swine herd sample collection data. [file 13567_2019_705_MOESM1_ESM.docx]

| **Sow parity** | **Total piglets born** | **Pig age @ time of sampling** |
| --- | --- | --- |
| Diarrheic piglets | | |
| 1 | 12 | 2 Days |
| 1 | 12 | 3 Days |
| 1 | 14 | 3 Days |
| 1 | 10 | 4 Days |
| 1 | 16 | 4 Days |
| 1 | 16 | 4 Days |
| 1 | 11 | 2 Days |
| 4 | 16 | 5 Days |
| 5 | 16 | 4 Days |
| 1 | 19 | 7 Days |
| 4 | 17 | 5 Days |
| 1 | 14 | 5 Days |
| 1 | 17 | 6 Days |
| 1 | 2 | 5 Days |
| 4 | 16 | 10 Days |
| Gilts =11  Sows =3 |  | Range = 2-10 days  Mean = 4 days |
| Healthy piglets | | |
| 3 | 16 | 11 Days |
| 4 | 21 | 8 Days |
| 4 | 16 | 8 Days |
| 4 | 15 | 5 Days |
| 2 | 10 | 6 Days |
| 5 | 18 | 4 Days |
| 4 | 19 | 4 Days |
| 4 | 16 | 4 Days |
| 4 | 17 | 5 Days |
| 3 | 17 | 6 Days |
| 5 | 18 | 4 Days |
| 5 | 17 | 5 Days |
| 5 | 19 | 4 Days |
| 2 | 12 | 3 Days |
| 4 | 20 | 3 Days |
| Gilts = 0  Sows = 15 |  | Range = 3-11 days  Mean = 5.3 days |

Number of litters (*n* = 4 pigs/litter) of diarrheic (fecal score 2-3) or healthy piglets (fecal score 0-1). Fecal samples based on fecal sample consistency scores (0=normal, 1=pasty, 2= liquid and 3=watery diarrhea) obtained from Cooper Farm in Ohio and the parity number of the sow/gilt to which they were born. Milk and serum from gilts (*n* = 11) and sows (*n* = 19) were collected on the day rectal swabs from piglets were collected. All samples were collected on 16^th^ of April, 2018.
